# Robust Trajectory Tracking Control for Continuous-Time Nonlinear Systems with State Constraints and Uncertain Disturbances

**DOI:** 10.3390/e24060816

**Published:** 2022-06-11

**Authors:** Chunbin Qin, Xiaopeng Qiao, Jinguang Wang, Dehua Zhang

**Affiliations:** School of Artificial Intelligence, Henan University, Zhengzhou 450000, China; qcb@henu.edu.cn (C.Q.); qxp@henu.edu.cn (X.Q.); wjg@henu.edu.cn (J.W.)

**Keywords:** adaptive dynamic programming, robust tracking control, control barrier function, state constraints

## Abstract

In this paper, a robust trajectory tracking control method with state constraints and uncertain disturbances on the ground of adaptive dynamic programming (ADP) is proposed for nonlinear systems. Firstly, the augmented system consists of the tracking error and the reference trajectory, and the tracking control problems with uncertain disturbances is described as the problem of robust control adjustment. In addition, considering the nominal system of the augmented system, the guaranteed cost tracking control problem is transformed into the optimal control problem by using the discount coefficient in the nominal system. A new safe Hamilton–Jacobi–Bellman (HJB) equation is proposed by combining the cost function with the control barrier function (CBF), so that the behavior of violating the safety regulations for the system states will be punished. In order to solve the new safe HJB equation, a critic neural network (NN) is used to approximate the solution of the safe HJB equation. According to the Lyapunov stability theory, in the case of state constraints and uncertain disturbances, the system states and the parameters of the critic neural network are guaranteed to be uniformly ultimately bounded (UUB). At the end of this paper, the feasibility of the proposed method is verified by a simulation example.

## 1. Introduction

With the continuous application of automatic driving technology [1,2] and intelligent robot technology [3,4], the role of the safety-critical system has attracted extensive attention. In the process of designing a controller, safety is the primary consideration compared to other performances. For the control system with strict safety requirements, the CBF was applied to the control system as a tool to achieve the purpose of state constraints.

Reinforcement learning (RL) can be regarded as the technology of strategy learning and evaluation learning. In the actual engineering application, although the phenomenon of the dimension curse exists in dynamic programming, RL can deal with it well, and we also call it adaptive dynamic programming (ADP) [5,6,7]. Adaptive dynamic programming is an intelligent control method, and it is also an approximate tool to deal with optimal control problems. However, the analytical solution of the Hamilton–Jacobi–Bellman (HJB) equation is generally difficult to obtain; therefore, the adaptive dynamic programming (ADP) can learn the solution of the HJB equation online by the neural network (NN) approximation method [8,9,10]. At present, a variety of control methods based on ADP have been proposed by researchers to deal with the problem of trajectory tracking and optimal control [11,12,13,14,15,16].

Adaptive dynamic programming enables the complex nonlinear system to achieve the desired tracking control goal [17,18,19,20]. In reference [17], the tracking performance of continuous-time nonlinear systems was analyzed by considering the influence of input constraints. Due to the influence of the actual situation, a series of uncertain disturbance factors are often considered. Therefore, robust optimal tracking control has become a research hot spot. In reference [18], solving tracking problems for complex nonlinear systems with uncertainty can be more difficult, and the adaptive criticism technique was used to solve the robust tracking control problems of nonlinear systems with random disturbances. Considering the nonlinear system with a continuous-time matching uncertainty, an effective robust tracking control method was adopted, and the discounted coefficient was selected for the nominal augmented error system in references [19,20]. Considering the system with disturbances, $H_{\infty }$ tracking control was used in control systems with disturbances [21]. In order to reduce the design cost and waste of resources and adjust the accuracy of the control system, a tracking control method based on the event triggering was proposed [22]. Considering the optimal regulation problem, a new non-quadratic discount performance function was proposed in reference [23]. In reference [24], an improved adaptive robust tracking method was proposed for the uncertainty of nonlinear systems and successfully extended to the mass-spring-damper system. The tracking control method proposed above enabled the feasibility of the control strategy and enabled the system to achieve the predetermined control target. However, none of the tracking control methods proposed above consider the state constraints problem.

In references [25,26,27,28], different ADP-based methods were proposed to solve various engineering problems. In some specific environments, the control system is often required to have reliable security. The purpose for which the safety system was designed is to find its control strategy by conforming to the safety specifications specified by the physical constraints of the system [29]. The use of the CBF method to solve the safety constraints of systems with strict requirements has attracted extensive attention [30,31,32,33]. Let the states displayed by the system converge to the desired equilibrium point; an approximate adjustment method for solving the optimization problem of safety boundary control was proposed, and the cost of violating the safety constraint was directly embedded into the value function [34]. In reference [35], the application of the CBF was introduced, and the verification method and the characteristics of implementation safety in the context of a safety-critical control system are summarized. The discrete-time state constraints problem was described in reference [36], and the HJB equation with the CBF was solved by using the approximate properties of the neural network.

In this paper, a new guaranteed cost robust tracking method with state constraints and uncertain disturbances is proposed. This method can guarantee the convergence of the system error under conditions of uncertain disturbances and state constraints. The discounted coefficient is selected for the nominal augmented system with tracking errors. In addition, the CBF is added to the system to solve the constraint problem of system states. Finally, the approximation property of the critic NN is adopted to deal with the HJB equation. The contributions of this paper are described below:

1. For robust tracking control problems, the CBF is applied to the tracking control system with uncertain disturbances so that the system can still have good tracking performance in the case of state constraints;
2. Combining the traditional adaptive control method with the CBF, the CBF is directly extended to the original system, and the CBF is used as a penalty function to punish unsafe behavior;
3. A new guaranteed cost robust adaptive tracking method with state constraints and uncertain disturbances is proposed to solve the safety HJB equation through the critic NN learning framework, and the critic NN parameters are guaranteed to be uniformly ultimately bounded (UUB) under the influence of state constraints and uncertain disturbances.

The arrangement of other parts of this article is described below: Section 2 states the preliminary knowledge and introduces the relevant contents of the control barrier function. Section 3 describes the selection of discount value functions for the nominal augmented system and introduces the form of the new cost function after adding the barrier function. Section 4 introduces the learning method of a critic neural network with state constraints and uncertain disturbances. In Section 5, the effectiveness of the proposed method is verified by a simulation example. Finally, some conclusions are summarized in Section 6.

## 2. Preliminaries

### 2.1. Problem Statement

Consider the following uncertain nonlinear safety system

(1) $$\dot{x}\left(t\right)=f\left(x\left(t\right)\right)+g\left(x\left(t\right)\right)u\left(t\right)+\Delta f\left(x\left(t\right)\right),$$

where x(t)∈Φ⊂$R^{n}$ is the state variable, u(t)∈U⊂$R^{m}$ is the control vector, Φ represents the safe feasible states set, U represents all admissible input sets, f(x(t))∈$R^{n}$ and g(x(t))∈$R^{m}$ are known functions with f(0)=0, and Δf(x(t))∈$R^{n}$ is the unknown perturbation term with Δf(0)=0. Here, let the initial state $x\left(0\right)=x_{0}$; we assume that there exists a constant $g_{M}$ and it satisfies 0<∥g(x)∥$\leq g_{M}$ for $\forall x\in R^{n}$, and Δf(x)=g(x)d(x), where $d\left(x\right)\in R^{m}$ is an unknown perturbation, and the known perturbation function $d_{M}\left(x\right)>0$ is the boundary of d(x), here, $∥d\left(x\right)∥\leq d_{M}\left(x\right)$. In addition, d(0)=0 and $d_{M}\left(0\right)=0$.

**Assumption** **1.** *Let the reference trajectory of the system *(1)* be $x_{d}\left(t\right)$, and $x_{d}\left(t\right)$ is a bounded function, which is limited and generated by the command generator ${\dot{x}}_{d}\left(t\right)=r\left(x_{d}\left(t\right)\right)$. Meanwhile, the reference trajectory $x_{d}\in R^{n}$ and the command function $r\in R^{n}$ are all Lipschitz continuous.*

### 2.2. Control Barrier Function

The application of the CBF further solves the constraint problem of the system [36]. In a predefined security set, the CBF candidate is always positive and tends to infinity at the defined set boundary. The CBF has a negative derivative at infinity, so the CBF will not reach infinity. If the state of the system is close to the safety boundary, then the condition that the derivative is negative will return the state to the safety set, so that the state displayed by the system will be maintained within the predetermined set. The safe feasible set Φ consists of operational constraints and safety specifications [34],

(2) $$\Phi =\{x\in R^{n}|h\left(x\right)\geq 0\},$$

(3) $$\partial \Phi =\{x\in R^{n}|h\left(x\right)=0\},$$

(4) $$Int\Phi =\{x\in R^{n}|h\left(x\right)>0\},$$

where ∂Φ represents the boundary of the safe feasible set Φ, IntΦ represents the interior of the set Φ, and *h* is a continuously differentiable function of *x*, which is composed of a one-dimensional system constraint range.

The CBF candidate B(x) satisfies all of the following properties,

(5) $$\frac{1}{\alpha_{1}\left(h\left(x\right)\right)}\leq B\left(x\right)\leq \frac{1}{\alpha_{2}\left(h\left(x\right)\right)},\forall x\in int\Phi$$

(6) $$\dot{B}\left(x\right)\leq \alpha_{3}\left(h\left(x\right)\right),\forall x\in int\Phi$$

where $\alpha_{1}\left(\cdot \right)$, $\alpha_{2}\left(\cdot \right)$, and $\alpha_{3}\left(\cdot \right)$ are Lipschitz class K functions, and B(x) is a control barrier function.

**Assumption** **2.** *Under the condition of uncertain disturbances, to ensure that the states of the system are constrained. We use a logarithmic control barrier function $B_{r}\left(x\right)$, which satisfies the following properties,*

(7) $$\left\{\begin{array}{c} B_{r}\left(x\right)>0,\forall x\in \Phi ,\\ B_{r}\left(x\right)\to \infty ,\forall x\in \partial \Phi . \end{array}\right.$$

*Besides, $B_{r}\left(x\right)$ is monotonically decreasing for* ∀x∈Φ.

Under the condition of satisfying Assumption 2, the expression of the specific logarithmic barrier function can be defined as

(8) $$B_{r}\left(x\right)=-log\left(\frac{\gamma h(x)}{\gamma h(x)+1}\right).$$

In (8), the parameter γ is a constant, and γ also determines the speed at which $B_{r}\left(x\right)$ is limited as it approaches the safety barrier.

Before describing the modified robust tracking method with constraints, we first make the following definitions and assumptions.

**Definition** **1.** *The safety control input set of the nonlinear system *(1)* is given below*

(9) $$U_{c}=\left\{u\in R^{m}|x^{u}\in int\Phi \right\},$$

*where $x^{u}$ is the system state associated with the control strategy u, and int*Φ* is the interior of the set defined in (4).*

**Assumption** **3.** *The initial condition of the nonlinear system (1) is strictly within *Φ*; in other words, $x_{0}\in int\Phi$. Assume that the initial set of allowed inputs is not empty and satisfies $U_{a}=U\cap U_{c}$. In addition, the control strategy $u\left(x_{0}\right)\in U_{a}$ exists.*

## 3. Guaranteed Cost Robust Tracking Design with State Constraints and Uncertain Disturbances

### 3.1. Modified Robust Adaptive Tracking Control

The augmented system is constructed by combining the tracking error and the reference trajectory. Before describing the modified robust adaptive tracking control, the tracking error is written as $e_{x}\left(t\right)=x\left(t\right)-x_{d}\left(t\right)$. According to (1), the tracking error system is derived as

(10) $$\begin{array}{c} {\dot{e}}_{x}\left(t\right)=f\left(x_{d}\left(t\right)+e_{x}\left(t\right)\right)+g\left(x_{d}\left(t\right)+e_{x}\left(t\right)\right)u\left(t\right)+\Delta f\left(x_{d}\left(t\right)+e_{x}\left(t\right)\right)-r\left(x_{d}\left(t\right)\right), \end{array}$$

where $r(x_{d}\left(t\right))$ is a Lipschitz continuous function.

By considering the tracking error dynamics (10), the infinite horizon cost function is given below [37]

(11) $$\begin{array}{c} \bar{V}\left(e_{x}\left(t\right),u\right)=\int_{t}^{\infty }e^{-\alpha (\tau -t)}U\left(e_{x}\left(\tau \right),u\left(\tau \right)\right)d\left(\tau \right), \end{array}$$

where α>0 is a discount factor, and $U(e_{x},u)$$=e_{x}^{T}Qe_{x}+u^{T}Ru$, both $Q\in R^{n\times n}$ and $R\in R^{m\times m}$ are symmetric positive definite matrices.

Under the condition of state constraints and uncertain disturbances, the purpose of dealing with the guaranteed cost tracking problem is to find the control input $u=u(e_{x}\left(t\right),x_{d}\left(t\right))$ and a positive real number ${\bar{V}}^{*}$; then the tracking error $e_{x}\left(t\right)$ converges to zero. Meanwhile, the cost function described in (11) satisfies $\bar{V}<{\bar{V}}^{*}$. It should be pointed out that ${\bar{V}}^{*}$ is called a guaranteed cost function, and the control *u* is called a guaranteed cost control input.

**Remark** **1.** *The discount term $e^{-\alpha (\tau -t)}$ given in *(11)* is mainly used to ensure that the cost function is $\bar{V}<\infty$ since the control $u(e_{x}\left(t\right),x_{d}\left(t\right))$ contains a part depending on the reference trajectory $x_{d}\left(t\right)$. In the absence of the discount term, $u(e_{x}\left(t\right),x_{d}\left(t\right))$ may make *(11)* to be unbounded. If the reference trajectory $x_{d}\left(t\right))$ does not converge to zero, the cost function *(11)* is unbounded without considering the discount term $e^{-\alpha (\tau -t)}$.*

Let $s\left(t\right)={\left[e_{x}^{T}\left(t\right),x_{d}^{T}\left(t\right)\right]}^{T}$∈$R^{2n}$, and the augmented system of error dynamics can be given

(12) $$\dot{s}\left(t\right)=F\left(s\left(t\right)\right)+G\left(s\left(t\right)\right)u\left(t\right)+\Delta F\left(s\left(t\right)\right),$$

the specific forms of F(s(t)) and G(s(t)) can be expressed as

$$F\left(s\left(t\right)\right)=\left[\begin{array}{c} f\left(x_{d}\left(t\right)+e_{x}\left(t\right)\right)-r\left(x_{d}\left(t\right)\right)\\ r(x_{d}\left(t\right)) \end{array}\right]$$

and

$$G\left(s\left(t\right)\right)=\left[\begin{array}{c} g(x_{d}\left(t\right)+e_{x}\left(t\right))\\ 0 \end{array}\right],$$

and ΔF(s(t))=G(s(t))d(s(t)), and because we know that $∥d\left(x\right)∥\leq d_{M}\left(x\right)$, it is very easy to know that the uncertain disturbance term $∥d\left(s\left(t\right)\right)∥\leq d_{M}\left(s\left(t\right)\right)$ holds, and $d_{M}\left(s\left(t\right)\right)$ is the boundary of the uncertain disturbance term d(s(t)).

**Remark** **2.** *There is a random disturbance term d(s(t)) in the augmented system *(12)* described above, which makes the process of designing the controller difficult. In the following introduction, the augmented error system *(12)* is equivalent to the optimal control of its nominal system, and the tracking problem with the random disturbance is transformed into an optimization adjustment problem with the discounted value function.*

Considering the existence of the uncertain term d(s(t)), the nominal system description of the system (12) is

(13) $$\dot{s}\left(t\right)=F\left(s\left(t\right)\right)+G\left(s\left(t\right)\right)u\left(t\right).$$

Inspired by references [29,36], $B_{r}\left(x\right)$ is combined with the nominal augmented system (13), and the modified value function is

(14) $$\begin{array}{c} V\left(s\left(t\right)\right)=\int_{t}^{\infty }e^{-\alpha (\tau -t)}\left[\rho d_{M}^{2}\left(s\left(\tau \right)\right)+s^{T}\left(\tau \right)Q_{T}s\left(\tau \right)+u^{T}\left(\tau \right)Ru\left(\tau \right)+B_{r}\left(s\left(\tau \right)\right)\right]d\left(\tau \right), \end{array}$$

where $Q_{T}$=$diag\{Q,0_{n\times n}\}$, $\rho =\lambda_{max}\left(R\right)$, the maximum eigenvalue of *R* can be expressed by $\lambda_{max}\left(R\right)$, both $Q\in R^{n\times n}$ and $R\in R^{m\times m}$ are weighted symmetric positive definite matrices of augmented systems, and α>0 is a discount coefficient.

According to Bellman’s principle of the optimal control theory [38], the minimum value of the Hamiltonian of the modified value function (14) of the nominal system (13) is given

(15) $$\begin{array}{c} H_{min}\left(s,u,V_{s}\right)=\rho d_{M}^{2}\left(s\right)+s^{T}Q_{T}s+u^{T}Ru-\alpha V\left(s\right)+B_{r}\left(s\right)+V_{s}^{T}\left(F\left(s\right)+G\left(s\right)u\right), \end{array}$$

where $V_{s}=\partial V/\partial s$, and the cost function $V^{*}\left(s\left(t\right)\right)$ can be considered as

(16) $$V^{*}\left(s\left(t\right)\right)=\min_{u}V\left(s\left(t\right)\right).$$

For the system (13) with the control barrier value function (14), since the equation $\partial H\left(s,u^{*},V_{s}^{*}\right)/\partial u^{*}=0$ holds, we can obtain the optimal control input $u^{*}$ from (15)

(17) $$u^{*}=-\frac{1}{2}R^{-1}G^{T}\left(s\right)V_{s}^{*},$$

where $V_{s}^{*}=\partial V^{*}\left(s\right)/\partial s$, and $V^{*}\left(s\right)$ denotes the optimal value V(s).

### 3.2. State Constraints Analysis

In the process of designing a robust tracking controller, the CBF as a constraint tool makes the states of the system evolve within the specified constraints, and the system can maintain good performance within the set safety constraints. The CBF provides a constraint tool for safety-critical systems to optimize the performance of other control objectives and clearly explains the priority of security compared to other performance indexes. In order to further describe that the CBF is bounded, it is described below that the boundedness of the CBF is demonstrated by changing the order of the controller.

**Lemma** **1.** *Consider an admissible feedback control strategy $u_{1}\in U_{a}$; there is the following time-invariant positive definite function Z, which satisfies $Z\in N^{1}$*

(18) $$\begin{array}{c} \frac{\partial V^{T}}{\partial x}\left(F\left(s\right)+G\left(s\right)u_{1}\right)+\rho d_{M}^{2}\left(s\right)+s^{T}Q_{T}s+u_{1}^{T}Ru_{1}-\alpha V\left(s\right)+B_{r}\left(s\right)=0, \end{array}$$

(19) $$V\left(s_{0},u_{1}\right)=Z\left(s_{0},u_{1}\right),$$

*where V is the value function of the system for all t∈[0,∞), and the following formula holds*

(20) V(s,u)=Z(s,u).

**Proof.**  Assume $V(s,u_{1})>0$ exists and is continuously differentiable; then, we have

(21) $$\begin{array}{cc} V\left(s\left(t\right),u_{1}\right)-V\left(s_{0},u_{1}\right)= & \int_{0}^{t}\dot{V}\left(s\left(\tau \right),u_{1}\right)d\left(\tau \right)=\int_{0}^{t}\frac{\partial V}{\partial s}\left(F+Gu_{1}\right)d\left(\tau \right). \end{array}$$

Considering (21), there are also

(22) $$\begin{array}{cc} Z\left(s\left(t\right),u_{1}\right)-Z\left(s_{0},u_{1}\right)= & -\int_{0}^{t}P\left(s\left(\tau \right),u_{1}\right)d\left(\tau \right), \end{array}$$

where $P\left(s,u\right)=\rho d_{M}^{2}\left(s\right)+s^{T}Q_{T}s+u^{T}Ru-\alpha V\left(s\right)+B_{r}\left(s\right)$.

We can derive from (21) and (22)

(23) $$\begin{array}{cc} Z\left(s\left(t\right),u_{1}\right)-V\left(s\left(t\right),u_{1}\right)= & \int_{0}^{t}\left(-\frac{\partial V}{\partial s}\left(F+Gu_{1}\right)-P\left(s\left(\tau \right),u_{1}\right)\right)d\left(\tau \right)+Z\left(s_{0},u_{1}\right)-V\left(s_{0},u_{1}\right). \end{array}$$

Combining (18), (21), and (23), we can obtain

(24) $$\begin{array}{cc} Z\left(s\left(t\right),u_{1}\right)-V\left(s\left(t\right),u_{1}\right)= & \int_{0}^{t}\left(P\left(s\left(\tau \right),u_{1}\right)-P\left(s\left(\tau \right),u_{1}\right)\right)d\left(\tau \right)=0. \end{array}$$

Therefore, we can obtain

(25) $$Z\left(s\left(t\right),u_{1}\right)=V\left(s\left(t\right),u_{1}\right).$$

This completes the proof. □

**Lemma** **2.** *We consider a series of positive definite value functions $V(s,t,u_{1})$, $V(s,t,u_{2})$, …, and $V(s,t,u_{i})$, and the corresponding abbreviations are $V_{1}$, $V_{2}$, …, and $V_{k}$, which are concerned with the allowable control inputs $u_{1}\left(s,t\right)$, $u_{2}\left(s,t\right)$, …, and $u_{k}\left(s,t\right)\in U_{a}$. Then, the Hamiltonian value defined in *(15)* satisfies the following conditions*

(26) $$H_{min_{1}}\leq H_{min_{2}}\leq \ldots H_{min_{i}},$$

*and the CBF candidate $B_{r}^{k}$ is bounded in the range of 1<k<i.*

**Proof.**  Assume that 0≤k≤j≤i is satisfied for any *j* and *k*, and the condition $H_{min_{k}}\leq H_{min_{j}}$ holds; therefore, one has

(27) $$V_{j}=V_{k}+V_{o},$$

where $V_{o}=V_{o}\left(s\left(t\right),u_{k}\right)$. According to (17), $u^{*}$ can be rewritten as

$$u^{*}=-\frac{1}{2}R^{-1}G^{T}\nabla V_{j},$$

$$\begin{array}{cc} H_{min_{j}}= & \nabla V_{j}^{T}\left(F+G\left(-\frac{1}{2}R^{-1}G^{T}\nabla V_{j}\right)\right)+T\left(s\right)+\frac{1}{4}\nabla V_{j}^{T}GR^{-1}G^{T}\nabla V_{j}. \end{array}$$

Considering $T\left(s\right)=\rho d_{M}^{2}\left(s\right)+s^{T}Q_{T}s+B_{r}\left(s\right)-\alpha V\left(s\right)$. According to (27), one may obtain

$$\begin{array}{c} H_{min_{j}}=H_{min_{k}}+\nabla V_{o}^{T}\left(F+Gu_{k}^{*}\right)-\left(u_{o}^{*T}Ru_{o}^{*}\right). \end{array}$$

According to the above description, since $H_{min_{j}}-H_{min_{k}}+\left(u_{o}^{*T}Ru_{o}^{*}\right)\geq 0$, we can obtain

$$\frac{\partial V_{o}\left(s,u_{k}\right)}{\partial t}\geq 0.$$

Because lim${}_{t\to \infty }V_{o}\left(s\left(t\right)\right)=0$, the following results are obtained

$$V_{o}\leq 0,$$

$$V\left(s,t,u_{1}\right)>V\left(s,t,u_{2}\right)>\ldots >V\left(s,t,u_{i}\right).$$

From the above Lemmas 1 and 2, we can obtain

(28) $$Z\left(s\left(t\right),u_{k}\right)<Z\left(s\left(t\right),u_{1}\right),1<k<i.$$

In the above derivation, not only $Z(s\left(t\right),u_{k})$ is bounded, but also P(s,u) is positive definite, and then $B_{r}^{k}$ is also bounded. In other words, in the case of state constraints, the system states will not reach the safety boundary in the process of tracking the reference trajectory. This proves that the CBF is bounded within each moment. □

**Theorem** **1.** *For the performance optimization problem described in *(16)*, let both Assumption 2 and Assumption 3 hold. Through the improvement of control input *(17)*, the security of the tracking state is guaranteed within a certain range for all t>0.*

**Proof.**  Through the introduction to Lemmas 1 and 2 above, the performance functions $Z(s,u_{k})$ and candidate function $B_{r}^{k}$ are bounded at each moment after the control input (17) is changed. From Assumptions 1 and 2, at the boundary of the constraint range, the value of the barrier function $B_{r}^{k}$ will reach infinity; in other words, the CBF remains is bounded at any moment, which ensures that the states of the system never reach the safe boundary. □

In the above introduction, the CBF is directly added to the cost function, which makes the states of the system constrained. This method is applicable to the guaranteed cost robust trajectory tracking control without initial admissible control. The traditional tracking controller usually needs the initial admissible control law. Although the appropriate initial admissible control law is found, the appropriate initial admissible control law may not satisfy the condition of state constraints.

Due to the existence of the discount term $e^{-\alpha (\tau -t)}$ in Equation (11), to guarantee the stability of the closed-loop system in the process of the tracking reference trajectory process, a guaranteed cost adaptive critic NN learning framework is designed. Before proceeding to the next step, we make the following assumption.

**Assumption** **4.** Let $J_{1}\left(s\right)$ be a candidate of Lyapunov function and satisfy the condition of ${\dot{J}}_{1}\left(s\right)=\nabla J_{1}^{T}\left(s\right)\left(F\left(s\right)+G\left(s\right)u^{*}\right)<0$, and $J_{1}\left(s\right)$ is continuously differentiable, where $\nabla J_{1}\left(s\right)=\partial J_{1}\left(s\right)/\partial s$. Assume there exists a symmetric positive definite matrix Λ(s), and the condition of expression ${\left(\nabla J_{1}\left(s\right)\right)}^{T}\left(F\left(s\right)+G\left(s\right)u^{*}\right)=-{\left(\nabla J_{1}\left(s\right)\right)}^{T}\Lambda \left(s\right)\nabla J_{1}\left(s\right)$ holds.

## 4. Design of Guaranteed Cost Adaptive Critic NN Learning Framework

In this section, the approximation property of the critic NN is used to approximate the solution of the safety HJB Equation (15), a guaranteed cost adaptive critic NN learning framework is proposed, the weight of the critic NN is updated through online the learning scheme, and all the vectors of the critic NN finally are guaranteed to be UUB. Considering the cost function described in (16), we design a critic NN to approximate the cost function $V^{*}\left(s\left(t\right)\right)$ and its partial derivative

(29) $$V^{*}\left(s\right)=W^{T}\phi \left(s\right)+\epsilon_{v}\left(s\right),$$

(30) $$\nabla V^{*}\left(s\right)={\left(\nabla \phi \left(s\right)\right)}^{T}W+\nabla \epsilon_{v}\left(s\right),$$

where *W*∈$R^{l}$ is the ideal vector of the critic neural network, the activation function of the critic NN can be expressed as $\phi \left(s\right)={\left[\varphi_{1}\;\varphi_{2}\;\varphi_{3}\;\cdot \cdot \cdot \;\varphi_{l}\right]}^{T}\in R^{l}$, *l* is the number of hidden-layer neurons, ∇ϕ(s) is denoted as the derivative of ϕ(s), the approximation error of the critic NN is denoted by $\epsilon_{v}\left(s\right)$, and $\nabla \epsilon_{v}\left(s\right)$ is the derivative of $\epsilon_{v}\left(s\right)$.

**Assumption** **5.** *The vector W of the critic NN is bounded by a positive constant, i.e., $∥W∥<W_{M}$, the activation function ϕ(s) and its derivative ∇ϕ(s), the critic NN error $\epsilon_{v}\left(s\right)$ and its derivative $\nabla \epsilon_{v}\left(s\right)$, are bounded, and satisfy $∥\phi \left(s\right)∥<\phi_{\varepsilon }$, $∥\nabla \phi \left(s\right)∥<\phi_{d\varepsilon }$, $∥\epsilon_{v}\left(s\right)∥<\epsilon_{\sigma }$, and $∥\nabla \epsilon_{v}\left(s\right)∥$$<\epsilon_{d\sigma }$, where $\phi_{\varepsilon }$, $\phi_{d\varepsilon }$, $\epsilon_{\sigma }$, and $\epsilon_{d\sigma }$ are positive constants.*

From Equations (15), (16), and (30), the approximate error of the safety HJB form is

(31) $$\begin{array}{c} \rho d_{M}^{2}\left(s\right)+s^{T}Q_{T}s+u^{*T}Ru^{*}-\alpha V^{*}\left(s\right)+B_{r}\left(s\right)+W^{T}\nabla \phi \left(s\right)\left(F\left(s\right)+G\left(s\right)u^{*}\right)+\epsilon_{v1}\left(s\right)=0, \end{array}$$

where $\epsilon_{v1}\left(s\right)=\nabla \epsilon_{v}^{T}\left(s\right)\left(F\left(s\right)+G\left(s\right)u^{*}\right)$.

Considering Equations (17) and (30), we can draw the following conclusion,

(32) $$u^{*}=-\frac{1}{2}R^{-1}G^{T}\left(s\right){\left(\nabla \phi \left(s\right)\right)}^{T}W+\epsilon_{v2},$$

where $\epsilon_{v2}=-\left(1/2\right)R^{-1}G^{T}\left(s\right)\nabla \epsilon_{v}$. At the same time, we substitute (32) into (31) and can obtain

(33) $$\begin{array}{c} \rho d_{M}^{2}\left(s\right)+s^{T}Q_{T}s-\alpha W^{T}\phi +B_{r}\left(s\right)+W^{T}\nabla \phi F-\frac{1}{4}W^{T}\nabla \phi \varrho {\left(\nabla \phi \right)}^{T}W-\omega =0, \end{array}$$

where $\varrho =G\left(s\right)R^{-1}G\left(s\right)$, and $\omega =\alpha \epsilon_{v}-{\left(\nabla \epsilon_{v}\right)}^{T}F+\left(1/4\right){\left(\nabla \epsilon_{v}\right)}^{T}\varrho \nabla \epsilon_{v}+\left(1/2\right)W^{T}\nabla \phi \varrho \nabla \epsilon_{v}$ is the approximation error.

We do not know the value of the ideal weight *W*; therefore, by using the critic NN to approximate the cost function $V^{*}\left(s\right)$ as

(34) $$\hat{V}\left(s\right)={\hat{W}}^{T}\phi \left(s\right),$$

where $\hat{W}$ denotes the estimated value of the ideal vector *W*, and $\hat{V}$ is the estimated value of the ideal cost function $V^{*}$. We can obtain the approximate HJB equation form from Equations (15) and (34)

(35) $$\begin{array}{c} \hat{H}\left(s,u,{\hat{V}}_{s}\right)=\rho d_{M}^{2}\left(s\right)+s^{T}Q_{T}s+u^{T}Ru-\alpha \hat{V}\left(s\right)+B_{r}\left(s\right)+{\hat{V}}_{s}^{T}\left(F\left(s\right)+G\left(s\right)u\right). \end{array}$$

Based on Equation (34), the control input $\hat{u}\left(s\right)$ can be approximated by

(36) $$\hat{u}\left(s\right)=-\frac{1}{2}R^{-1}G^{T}\left(s\right){\left(\nabla \phi \left(s\right)\right)}^{T}\hat{W}.$$

Through Equations (31) and (35), we define the HJB equation error caused by the critic NN in the approximation process as

(37) $$\begin{array}{c} \varepsilon =\rho d_{M}^{2}\left(s\right)+s^{T}Q_{T}s-\alpha {\hat{W}}^{T}\phi +B_{r}\left(s\right)+{\hat{W}}^{T}\nabla \phi F-\frac{1}{4}{\hat{W}}^{T}\nabla \phi \varrho {\left(\nabla \phi \right)}^{T}\hat{W}. \end{array}$$

The estimation error of the weights of the critic NN is defined as $\tilde{W}$, and we can obtain

(38) $$\tilde{W}=W-\hat{W}.$$

The HJB approximation error can be defined as

(39) $$\varepsilon =-{\tilde{W}}^{T}\xi +\frac{1}{4}{\tilde{W}}^{T}\nabla \phi \varrho {\left(\nabla \phi \right)}^{T}\tilde{W}+\omega ,$$

where $\xi =\nabla \phi (F\left(s\right)+G\left(s\right)\hat{u})-\alpha \phi \left(s\right)$. The Lyapunov function candidate $J_{1}\left(s\right)$ is shown in Assumption 4, and we take $\Pi (s,\hat{u})$ as an indicator function and define it as

(40) $$\Pi \left(s,\hat{u}\right)=\left\{\begin{array}{c} 0,\mathrm{if}\;J_{1}^{T}\left(s\right)\left(F\left(s\right)+G\left(s\right)\hat{u}\right)<0,\\ 1,\mathrm{else}. \end{array}\right.$$

We choose $\hat{W}$ to minimize the square residual $E=\left(1/2\right)\varepsilon^{T}\varepsilon$, and then we obtain the minimum value of the HJB approximation error ε. We use the gradient descent method as the critic vector adjustment optimization law

(41) $$\begin{array}{c} \dot{\hat{W}}=-\beta \bar{\xi }\left(L\left(s\right)+Y\left(s\right)+B_{r}\left(s\right)+\rho d_{M}^{2}\left(s\right)\right)+\frac{\beta }{2}\Pi \left(s,\hat{u}\right)\nabla \phi \varrho J_{1}\left(s\right)+\beta \left(\left(K_{1}\theta^{T}-K_{2}\right)+A\left(s\right)\right)\hat{W}, \end{array}$$

where $\bar{\xi }=\xi /{\left(1+\xi^{T}\xi \right)}^{2}$, $\theta =\xi /(1+\xi^{T}\xi )$, $L\left(s\right)={\hat{W}}^{T}\nabla \phi F-\alpha {\hat{W}}^{T}\phi +s^{T}Q_{T}s$, $Y\left(s\right)=-\left(1/4\right){\hat{W}}^{T}\nabla \phi \varrho {\left(\nabla \phi \right)}^{T}\hat{W}$, and $A\left(s\right)=\left(1/4\right)\nabla \phi \varrho {\left(\nabla \phi \right)}^{T}\hat{W}{\left(\theta /\left(1+\xi^{T}\xi \right)\right)}^{T}$, and β>0 is a learning rate that determines the convergence speed of the critic NN. $K_{1}$ and $K_{2}$ are two tuning parameters.

From the above description, it is deduced that the weight estimation error is

(42) $$\begin{array}{c} \dot{\tilde{W}}=-\beta \bar{\xi }\left({\tilde{W}}^{T}\xi +\tilde{Y}\left(s\right)-B_{r}\left(s\right)\right)-\frac{\beta }{2}\Pi \left(s,\hat{u}\right)\nabla \phi \varrho J_{1}-\beta \left(\left(K_{1}\theta^{T}-K_{2}\right)-\tilde{A}\left(s\right)\right)\left(W-\tilde{W}\right), \end{array}$$

where $\tilde{Y}\left(s\right)=-\left(1/4\right){\tilde{W}}^{T}\nabla \phi \varrho {\left(\nabla \phi \right)}^{T}\tilde{W}$, $\tilde{A}\left(s\right)=\left(1/4\right)\nabla \phi \varrho {\left(\nabla \phi \right)}^{T}\left(W-\tilde{W}\right){\left(\theta /\left(1+\xi^{T}\xi \right)\right)}^{T}$.

**Theorem** **2.** Consider the nominal system *(13)*, the modified value function *(15)*, and the tuning laws *(41)*. Only if all the above Assumptions 1–5 hold, then the critic NN error $\tilde{W}$, the system state x, and the control input $u^{*}$ are guaranteed to be UUB.

**Proof.**  Analyze the Lyapunov candidate function described below

(43) $$L\left(t\right)=V_{s}\left(s\left(t\right)\right)+\frac{1}{2}{\tilde{W}}^{T}\beta^{-1}\tilde{W}.$$

The result of deriving Equation (43) is shown as

(44) $$\begin{array}{cc} \dot{L}\left(t\right)=\; & {\dot{V}}_{s}\left(s\left(t\right)\right)+{\tilde{W}}^{T}\beta^{-1}\dot{\tilde{W}}\\ =\; & {\dot{L}}_{V}+{\dot{L}}_{W}. \end{array}$$

The first term ${\dot{L}}_{V}$ is

(45) $$\begin{array}{cc} {\dot{L}}_{V}=\; & {\tilde{W}}^{T}\nabla \phi \left(s\right)\left(F\left(s\right)+G\left(s\right)u\right)+\nabla \epsilon_{v}^{T}\left(s\right)\left(F\left(s\right)+G\left(s\right)u\right)\\ =\; & W^{T}\left(\nabla \phi \left(s\right)F\left(s\right)-\frac{1}{2}D_{1}\hat{W}\right)+\epsilon_{v1}\left(s\right)\\ =\; & W^{T}\nabla \phi \left(s\right)F\left(s\right)+\frac{1}{2}W^{T}D_{1}\left(W-\hat{W}\right)-\frac{1}{2}W^{T}D_{1}W+\epsilon_{v1}\left(s\right)\\ =\; & W^{T}\nabla \phi \left(s\right)F\left(s\right)+\frac{1}{2}W^{T}D_{1}\tilde{W}-\frac{1}{2}W^{T}D_{1}\left(s\right)W+\epsilon_{v1}\left(s\right)\\ =\; & W^{T}\sigma +\frac{1}{2}W^{T}D_{1}\tilde{W}+\epsilon_{v1}\left(s\right), \end{array}$$

where $\epsilon_{v1}\left(s\right)=\nabla \epsilon_{v1}^{T}\left(s\right)\left(F\left(s\right)-\frac{1}{2}G\left(s\right)R^{-1}G{\left(s\right)}^{T}\nabla \phi \left(s\right)\tilde{W}\right)$, σ=∇ϕ(s)(F(s)+G(s)u), and $D_{1}=\nabla \phi \varrho {\left(\nabla \phi \right)}^{T}$.

The second term ${\dot{L}}_{W}$ can be obtained by (41)

(46) $$\begin{array}{cc} {\dot{L}}_{W}=\; & {\tilde{W}}^{T}\beta^{-1}\dot{\tilde{W}}\\ =\; & {\tilde{W}}^{T}\beta^{-1}[-\beta \bar{\xi }\left({\tilde{W}}^{T}\xi +\tilde{A}\left(s\right)-B_{r}\left(s\right)\right)-\frac{\beta }{2}\Pi \left(s,\hat{u}\right)\nabla \phi \varrho J_{1}-\beta (\left(K_{1}\theta^{T}-K_{2}\right)\\ & -\tilde{A}\left(s\right))\left(W-\tilde{W}\right)]\\ =\; & {\tilde{W}}^{T}\left[-\bar{\xi }\left({\tilde{W}}^{T}\xi +\tilde{A}\left(s\right)-B_{r}\left(s\right)\right)-\frac{1}{2}\Pi \left(s,\hat{u}\right)\nabla \phi \varrho J_{1}-\left(\left(K_{1}\theta^{T}-K_{2}\right)-\tilde{A}\left(s\right)\right)\left(W-\tilde{W}\right)\right]\\ =\; & {\tilde{W}}^{T}\left[-\bar{\xi }\left(\left({\tilde{W}}^{T}\xi -\omega \right)-\frac{1}{4}{\tilde{W}}^{T}D_{1}\tilde{W}\right)-\left(\left(K_{1}\theta^{T}-K_{2}\right)-\frac{D_{1}}{4}\left(W-\tilde{W}\right)\frac{\theta^{T}}{m}\right)\left(W-\tilde{W}\right)\right]\\ & -\frac{1}{2}\Pi \left(s,\hat{u}\right){\tilde{W}}^{T}\nabla \phi \varrho J_{1}\\ =\; & {\tilde{W}}^{T}\left[-\theta {\tilde{W}}^{T}\theta +\frac{\theta \omega }{m}+\frac{\theta {\tilde{W}}^{T}D_{1}\tilde{W}}{4}\right]-{\tilde{W}}^{T}[\left(K_{1}\theta^{T}-K_{2}\right)\left(W-\tilde{W}\right)\\ & -\frac{D_{1}}{4}\left(W-\tilde{W}\right){\left(\theta /m\right)}^{T}\left(W-\tilde{W}\right)]-c, \end{array}$$

where $m=1+\xi^{T}\xi$, $c=-\frac{\Pi \left(s,\hat{u}\right){\tilde{W}}^{T}\nabla \phi \varrho J_{1}}{2}$. Further, we can obtain

(47) $$\begin{array}{cc} {\dot{L}}_{W}= & -{\tilde{W}}^{T}\theta {\tilde{W}}^{T}\theta +\frac{{\tilde{W}}^{T}\theta \omega }{m}+\frac{{\tilde{W}}^{T}\theta {\tilde{W}}^{T}D_{1}\tilde{W}}{4}-{\tilde{W}}^{T}\left(K_{1}\theta^{T}-K_{2}\right)\left(W-\tilde{W}\right)+\frac{{\tilde{W}}^{T}D_{1}\theta^{T}}{4m}(W\\ & -\tilde{W})\left(W-\tilde{W}\right)-c\\ = & -{\tilde{W}}^{T}\theta {\tilde{W}}^{T}\theta +\frac{{\tilde{W}}^{T}\theta \omega }{m}+\frac{{\tilde{W}}^{T}\theta {\tilde{W}}^{T}D_{1}\tilde{W}}{4}-{\tilde{W}}^{T}\left(K_{1}\theta^{T}-K_{2}\right)W+{\tilde{W}}^{T}\left(K_{1}\theta^{T}-K_{2}\right)\tilde{W}\\ & +\frac{{\tilde{W}}^{T}D_{1}W\theta^{T}}{4m}W-\frac{{\tilde{W}}^{T}D_{1}\tilde{W}\theta^{T}}{4m}W-\frac{{\tilde{W}}^{T}D_{1}W\theta^{T}}{4m}\tilde{W}+\frac{{\tilde{W}}^{T}D_{1}\tilde{W}\theta^{T}}{4m}\tilde{W}-c. \end{array}$$

Taking the sum of the terms ${\dot{L}}_{V}$ and ${\dot{L}}_{W}$, we obtain

(48) $$\begin{array}{cc} \dot{L}\left(t\right)=\; & W^{T}\sigma +\frac{1}{2}W^{T}D_{1}\tilde{W}-{\tilde{W}}^{T}\theta {\tilde{W}}^{T}\theta +\frac{{\tilde{W}}^{T}\theta \omega }{m}+\frac{{\tilde{W}}^{T}\theta {\tilde{W}}^{T}D_{1}\tilde{W}}{4}-{\tilde{W}}^{T}\left(K_{1}\theta^{T}-K_{2}\right)W\\ & +{\tilde{W}}^{T}\left(K_{1}\theta^{T}-K_{2}\right)\tilde{W}+\frac{{\tilde{W}}^{T}D_{1}W\theta^{T}}{4m}W-\frac{{\tilde{W}}^{T}D_{1}\tilde{W}\theta^{T}}{4m}W-\frac{{\tilde{W}}^{T}D_{1}W\theta^{T}}{4m}\tilde{W}\\ & +\frac{{\tilde{W}}^{T}D_{1}\tilde{W}\theta^{T}}{4m}\tilde{W}-c+\epsilon_{v1}\left(s\right). \end{array}$$

Assume that $Z={\left[{\tilde{W}}^{T}\theta ,{\tilde{W}}^{T}\right]}^{T}$, then we can obtain

$$\dot{L}\left(t\right)=-Z^{T}\left[\begin{array}{cc} I & -\frac{W^{T}D_{1}}{8m}-\frac{K_{1}^{T}}{2}\\ -\frac{D_{1}W}{8m}-\frac{K_{1}}{2} & K_{2}-\frac{\theta^{T}WD_{1}}{4m} \end{array}\right]Z$$

(49) $$+Z^{T}\left[\begin{array}{cc} \frac{\omega }{m} & \\ \frac{D_{1}W\theta^{T}W}{4m}+K_{2}W-K_{1}\theta^{T}W \end{array}\right]+b+d,$$

where

(50) $$b=c+W^{T}\sigma +∥\epsilon_{v1}\left(s\right)∥,$$

(51) $$d=\frac{W^{T}D_{1}\left(\tilde{W}\theta^{T}\tilde{W}-\tilde{W}\theta^{T}W-W\theta^{T}\tilde{W}\right)}{4m}.$$

Define

(52) $$M=\left[\begin{array}{cc} I & -\frac{W^{T}D_{1}}{8m}-\frac{K_{1}^{T}}{2}\\ -\frac{D_{1}W}{8m}-\frac{K_{1}}{2} & K_{2}-\frac{\theta^{T}WD_{1}}{4m} \end{array}\right],$$

(53) $$a=\left[\begin{array}{cc} \frac{\omega }{m} & \\ \frac{D_{1}W\theta^{T}W}{4m}+K_{2}W-K_{1}\theta^{T}W \end{array}\right].$$

Let the tuning parameters $K_{1}$, $K_{2}$, and γ be chosen so that M>0, and we obtain

(54) $$\dot{L}\left(t\right)<-{∥Z∥}^{2}\sigma_{min}\left(M\right)+∥a∥∥Z∥+\mu ,$$

where μ=b+d. In summary, the Lyapunov derivative $\dot{L}\left(t\right)$ is negative if

(55) $$∥Z∥>\frac{∥a∥}{2\sigma_{min}\left(M\right)}+\sqrt{\frac{\mu }{\sigma_{min}\left(M\right)}+\frac{a^{2}}{4\sigma_{min}\left(M\right)}}.$$

Based on the Lyapunov theorem [39], as long as the selected appropriately tuning parameters $K_{1}$, $K_{2}$, and γ make the formula (32) hold, in the case of state constraints and uncertain disturbances, the critic NN weight error $\tilde{W}$, the system state *x*, and the control input $u^{*}$ are guaranteed to be UUB, and the nonlinear system (1) is guaranteed to be closed-loop stable in the presence of state constraints and uncertain disturbances. The proof is completed. □

## 5. Simulation

We consider a spring-mass-damping system with nonlinear properties [22], and the system dynamics of the spring-mass-damper are as follows [24]

(56) $$\begin{array}{c} \left\{\begin{array}{c} {\dot{x}}_{1}=x_{2},\\ {\dot{x}}_{2}=-\frac{K(x_{2})}{M}-\frac{C}{M}x_{1}+\frac{1}{M}u+px_{1}sin\left(x_{2}\right), \end{array}\right. \end{array}$$

where $x={\left[x_{1},x_{2}\right]}^{T}\in R^{2}$ and the nonlinear condition $K\left(x\right)=x^{3}$, $x_{1}$, and $x_{2}$ are the position and velocity, respectively, and *u* is the force applied to the object. *M* is the mass of the object. *K* is the stiffness constant of the spring, and *C* is the damping. The above system dynamics parameters are M=1 kg and C=0.5 N· s/m. A mismatched disturbance may lead to system instability. Considering that the system still has stable performance under disturbances, the uncertain disturbance matching the system is selected, the uncertain disturbance term $d\left(x\right)=px_{1}sin\left(x_{2}\right)$, and we assume that p∈[−1,1] and $d_{M}\left(x\right)=∥x∥$.

In the simulation process, considering that the initial allowable control law is not required, to make the tracking errors of the system converge to zero, the reference trajectory gradually tending to zero is selected, and the following reference trajectory $x_{d}\left(t\right)$ is given

(57) $${\dot{x}}_{d}=\left[\begin{array}{c} -0.5x_{d1}-x_{d2}cos\left(x_{d1}\right)\\ sin\left(x_{d1}\right)-x_{d2} \end{array}\right],$$

the initial condition is given as $x_{d}\left(0\right)={\left[0.15,0.25\right]}^{T}$, and we set the augmented state vector as $s={\left[e_{x}^{T},x_{d}^{T}\right]}^{T}$, and then combine (56) with (57), the dynamics of the augmented system can be derived

(58) $$\dot{s}=\left[\begin{array}{c} s_{2}+s_{4}+0.5s_{3}+s_{4}cos\left(s_{3}\right)\\ -{\left(s_{2}+s_{4}\right)}^{3}-0.5\left(s_{1}+s_{3}\right)-sin\left(s_{3}\right)+s_{4}\\ -0.5s_{3}-s_{4}cos\left(s_{3}\right)\\ sin\left(s_{3}\right)-s_{4} \end{array}\right]+\left[\begin{array}{c} 0\\ 1\\ 0\\ 0 \end{array}\right]\left(u+d\left(s\right)\right),$$

where $s={\left[s_{1},s_{2},s_{3},s_{4}\right]}^{T}={\left[e_{x1},e_{x2},x_{d1},x_{d2}\right]}^{T}$ with $e_{xi}=x_{i}-x_{di}$.

To constrain the states of the system in augmented system dynamics (58), the control barrier function used is as follows

(59) $$\begin{array}{c} \left\{\begin{array}{c} B_{r1}\left(s_{1}+s_{3}\right)=-log\left(\frac{\gamma h(s_{1}+s_{3})}{\gamma h(s_{1}+s_{3})+1}\right)\\ B_{r2}\left(s_{2}+s_{4}\right)=-log\left(\frac{\gamma h(s_{2}+s_{4})}{\gamma h(s_{2}+s_{4})+1}\right). \end{array}\right. \end{array}$$

The state constraints of the system are given as $-0.2\leq x_{1}\leq 0.35$ and $-0.15\leq x_{2}\leq 0.4$, and the parameter γ=0.02.

To complete the design of robust trajectory tracking control, the modified value function (14) can be specified as

(60) $$\begin{array}{c} V\left(s\left(t\right)\right)=\int_{t}^{\infty }e^{-\alpha (\tau -t)}\left[\rho d_{M}^{2}\left(s\left(\tau \right)\right)+s^{T}\left(\tau \right)Q_{T}s\left(\tau \right)+u^{T}\left(\tau \right)Ru\left(\tau \right)+B_{r1}\left(s\left(\tau \right)\right)+B_{r2}\left(s\left(\tau \right)\right)\right]d\left(\tau \right). \end{array}$$

Besides, we select the learning rate as β=1.5 and the discount factor α = 0.15, respectively. In order to deal with the approximate optimal control for the nominal augmented part of (58), we choose $Q_{T}$ = $diag\{5I_{2},0_{2\times 2}\}$ and R=I, and *I* denotes an identity matrix of appropriate dimensions. In this example, the activation function for the critic NN is chosen as ϕ(s) = ${\left[s_{1}^{2},s_{1}s_{2},s_{1}s_{3},s_{1}s_{4},s_{2}^{2},s_{2}s_{3},s_{2}s_{4},s_{3}^{2},s_{3}s_{4},s_{4}^{2}\right]}^{T}$. In addition, the weights of the critic NN are denoted as ${\hat{W}}_{c}$ = ${\left[W_{c1},W_{c2},\ldots ,W_{c10}\right]}^{T}$. The initial value of the state is given as $x\left(0\right)={\left[-0.2,0.4\right]}^{T}$, and it is easy to calculate the initial error vector according to $s\left(0\right)=x\left(0\right)-x_{d}\left(0\right)$, so the initial state of the augmented system is $s\left(0\right)={\left[-0.35,0.15,0.15,0.25\right]}^{T}$. In order to satisfy the condition of persistency of excitation, an exploration noise $exp\left(-0.25t\right)sin^{2}\left(t\right)cos\left(t\right)$ is added during the training of the neural network.

**Figure 1:**
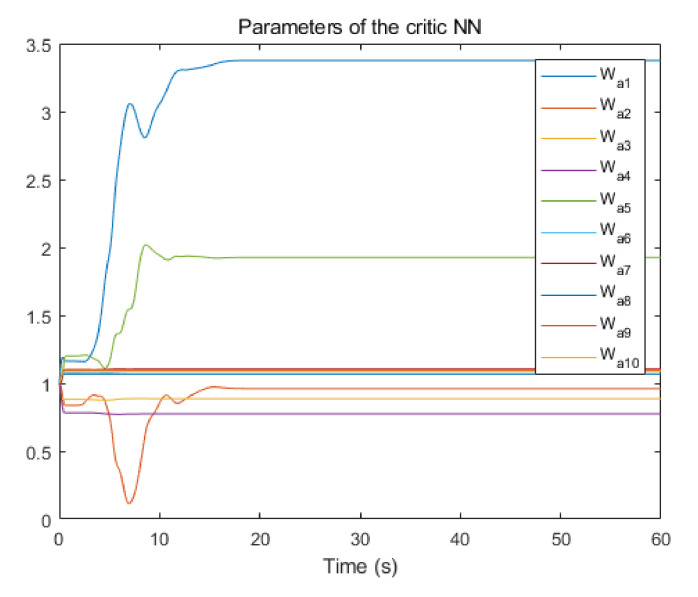
Convergence of parameters of the critic NN.

**Figure 2:**
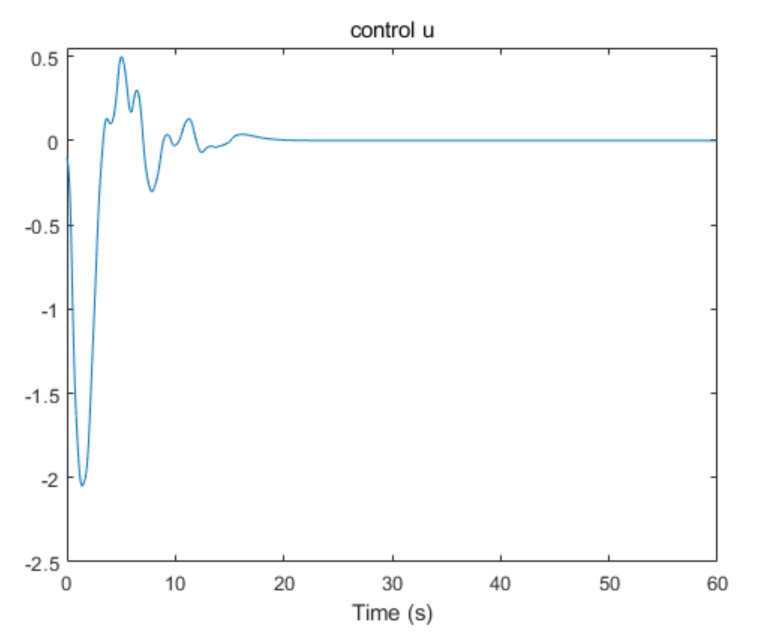
The control input of the system.

**Figure 3:**
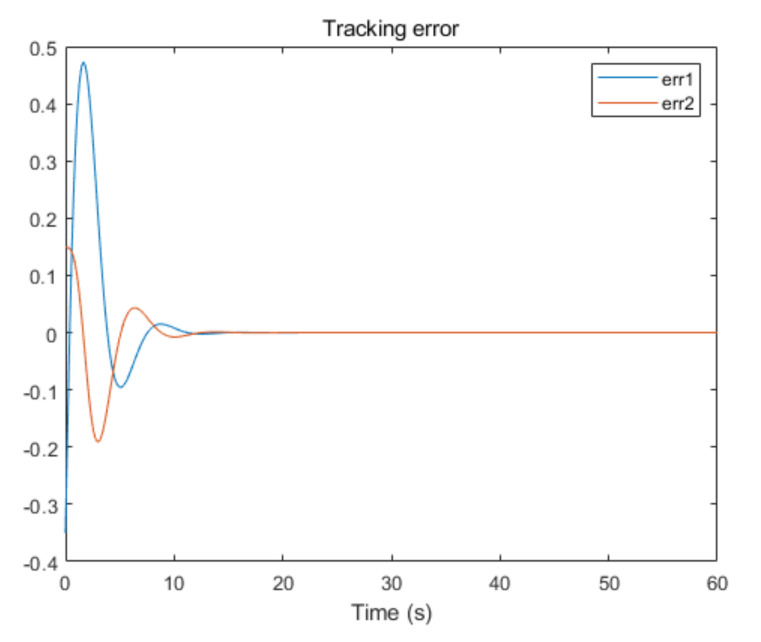
Tracking error of system without state constraints (p=0.8).

**Figure 4:**
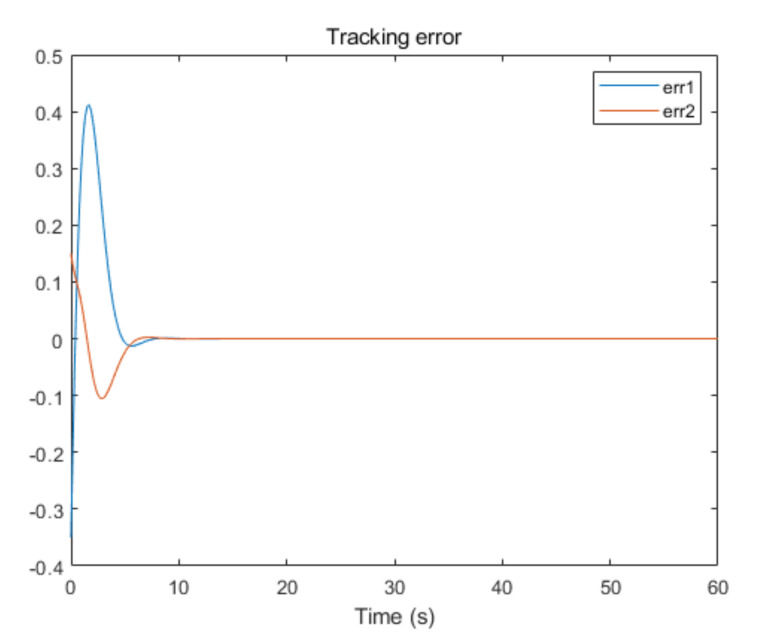
Tracking error of system with state constraints (p=0.8).

**Figure 5:**
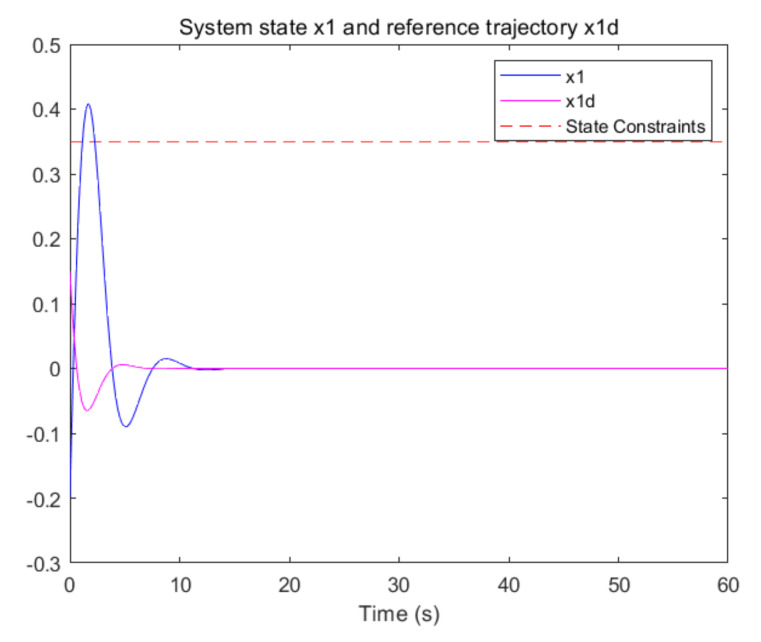
Trajectory of the system state $x_{1}$ without state constraints (p=0.8).

**Figure 6:**
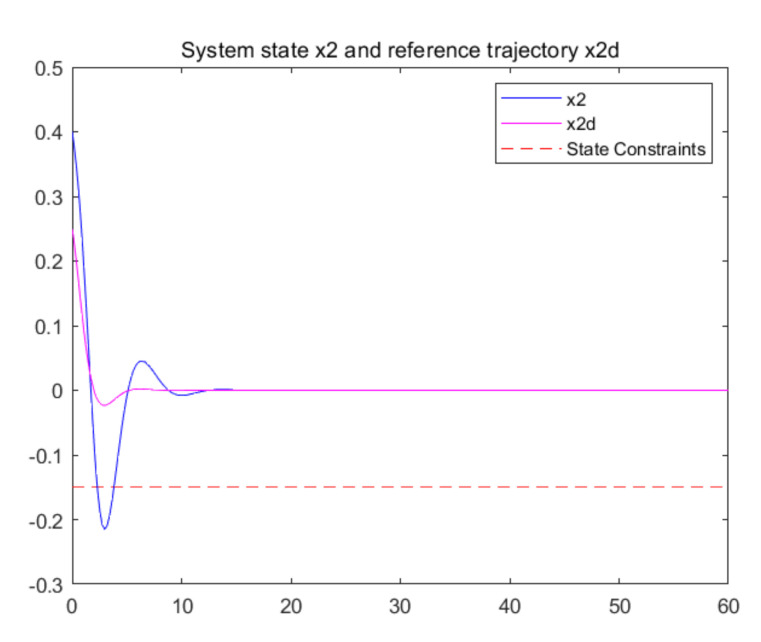
Trajectory of the system state $x_{2}$ without state constraints (p=0.8).

**Figure 7:**
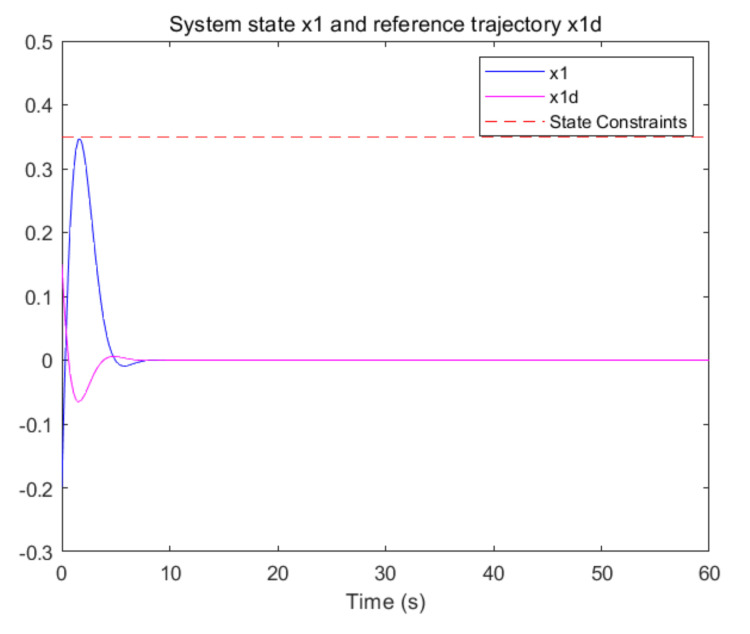
Trajectory of the system state $x_{1}$ with state constraints (p=0.8).

**Figure 8:**
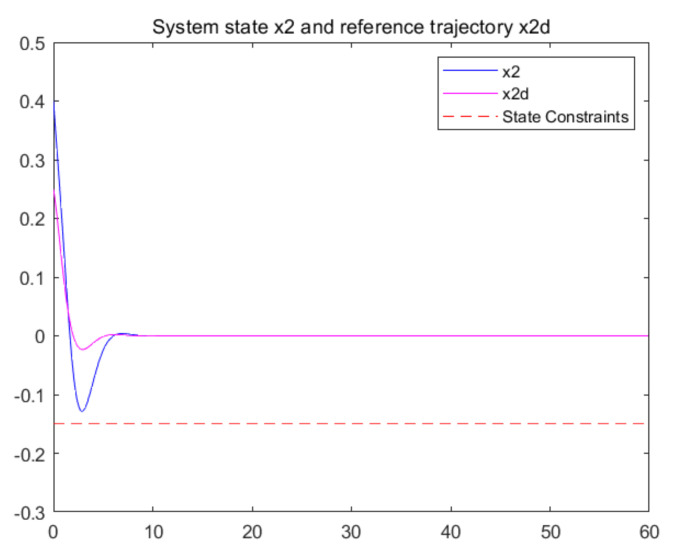
Trajectory of the system state $x_{2}$ with state constraints (p=0.8).

The convergence of critic parameters is shown in Figure 1, and the critic parameters after 30 s converge to $\hat{W}={\left[3.3767,0.9606,0.8867,0.7752,1.9266,1.0686,1.105,1.067,1.0992,1.0898\right]}^{T}$. Figure 2 shows the control inputs of the system. Figure 3 shows the trajectory of the tracking errors $e_{x1}$ and $e_{x2}$ of the system without state constraints. Figure 4 shows the tracking error of the system under state constraints. Figure 5 and Figure 6 show that the system tracks the reference trajectory without state constraints, and we can see that the system states violate the constraints. Figure 7 and Figure 8 show that the system tracks the desired trajectory with state constraints, and that under the condition of state constraints and uncertain disturbances, the system still maintains good performance. The method described in this paper can ensure the stability of the closed-loop system. In summary, the simulation results display the effectiveness of the proposed method.

## 6. Conclusions

This paper presented a robust trajectory tracking method for nonlinear systems with state constraints and uncertain disturbances based on adaptive dynamic programming. Firstly, the system error was combined with the reference trajectory to construct the augmented system, and at the same time, the nominal system of the augmented system was considered. In order to overcome the uncertain disturbances of the augmented system, the discount coefficient was introduced into the nominal system, and the CBF was added into the nominal system with the discount coefficient to constrain the states of the system. In addition, cost functions and control strategies were learned by designing a guaranteed cost adaptive critic NN learning framework. Finally, the simulation results demonstrated that the described method can converge the system error within the state constraints. In the next work, we will try to extend the state constraints method to discrete-time tracking control systems and multi-agent systems.

## Data Availability

The authors can confirm that all relevant data are included in the article.
